# Disappearance and Re-Emergence of Influenza during the COVID-19 Pandemic: Association with Infection Control Measures

**DOI:** 10.3390/v15010223

**Published:** 2023-01-13

**Authors:** Hikaru Takeuchi, Ryuta Kawashima

**Affiliations:** 1 Division of Developmental Cognitive Neuroscience, Institute of Development, Aging and Cancer, Tohoku University, Sendai 980-8575, Japan; 2 Smart Aging Research Center, Tohoku University, Sendai 980-8575, Japan; 3 Department of Advanced Brain Science, Institute of Development, Aging and Cancer, Tohoku University, Sendai 980-8575, Japan

**Keywords:** influenza, mask, social distancing, control measures

## Abstract

During the coronavirus disease 2019 (COVID-19) pandemic, the influenza virus had a very low prevalence, and in many areas, outbreaks were almost non-existent. In this study, the associations between infection control measures taken for COVID-19 and the global disappearance of the influenza virus were investigated. The detection rate of influenza from baseline was investigated during four seasons (12 weeks from epidemiological week 49 in 2020 and 2021 and 12 weeks from epidemiological week 23 in 2020 and 2021) in each country participating in the surveillance system of the World Health Organization. Three measures of infection control: mask use ratio, social distancing index (an index of human mobility and physical distance obligations), and an index of stringency of measures taken by authorities were studied. In mid-2020, most countries analyzed had high levels of infection control measures, and in most countries, influenza was drastically reduced compared to previous years. Multiple regression analyses compared the study data with data from other seasons. There was an association between high mask use with low influenza detection in all three remaining seasons, an association between a low social distancing index (low mobility and more social contact obligations) with a low influenza detection rate in two seasons, and a marginal significant association of high stringency index with a low influenza detection rate(in 2020-end-seasons). These results support the notion that seasonal influenza is controllable through effective preventive measures, especially those of mask use and human social contact, and these measures should be recommended during future waves of novel influenza virus infection.

## 1. Introduction

Influenza is a viral respiratory tract infection and is a major cause of morbidity and mortality worldwide. There are an estimated 3–5 million cases of severe influenza illness and 250,000–500,000 annual deaths worldwide [1]. Importantly, at the time of the coronavirus disease 2019 (COVID-19) pandemic, in many areas of the world, influenza outbreaks were almost non-existent during the pandemic. It is speculated that non-pharmacological interventions (NPIs), such as social distancing, lockdown, and the use of masks for COVID-19, were the cause [2]. It was also pointed out that the disappearance of such influenza could not be explained by viral interference, since the COVID-19 was spreading even in areas where it had not spread serologically [3]. However, in some areas, influenza returned relatively early in 2020 [4]. In the United States, influenza returned in the winter of 2021, and in Japan, influenza had not returned in the winter of 2021. It has been also pointed out that he reemergence of such influenza occurred after the NPI was greatly relaxed, suggesting that the relaxation of NPI was behind the reemergence of influenza [5].

However, to the best of our knowledge, there are no studies that show statistically what is the correlation between these regional differences in the global disappearance and return of influenza. The purpose of this study was to clarify these issues. The World Health Organizations’ (WHO) influenza surveillance system and publicly available NPI information were studied to identify the factors involved with influenza’s disappearance and return. 

Given the deaths that influenza causes annually around the world and the concern about new strains of influenza, it is important to reveal this issue.

## 2. Methods

### 2.1. Influenza Data

The WHO influenza surveillance data for the 12 weeks following epidemiologic week 23 and epidemiological week 49 were studied in each country from the World Health Organization Influenza Laboratory Surveillance Information [6], which is currently not working; the alternative site provides the same data [7]. These two periods were chosen because, in many countries, seasonal influenza outbreaks are seen only in these two periods (winter season in each hemisphere), even though in some countries, influenza outbreaks are seen throughout the year. The disappearance and re-emergence of influenza were evaluated in four periods: the 12 weeks from epidemiological week 49 in 2020 and 2021 and the 12 weeks from epidemiological week 23 in 2020 and 2021. The following equations were used: (1) 2 × (number of confirmed influenza cases in the 12 weeks from week 49 in 2020)/(the number of cases in that period in 2017 plus 2018). The season of 2019 was excluded because influenza had already been affected by the COVID-19 outbreak in some countries. (2) 2 × (number of confirmed influenza cases in the 12 weeks from week 49 in 2021)/(the number of cases in that period in 2017 plus 2018), (3) 2 × (number of confirmed influenza cases in the 12 weeks from week 23 in 2020)/(the number of cases in 2018 plus 2019), and (4) 2 × (number of confirmed influenza cases in the 12 weeks from week 23 in 2021)/(the number of cases in 2018 plus 2019).

Then, the log-transformation of this ratio is used to mitigate the non-linearity of the associations between a dependent variable and independent variables in multiple regression analyses. As the dependent variable contains many zeros (countries where no influenza was detected during the relevant period of the pandemic at all), the log transformation adds one-half of the value of the lowest non-zero dependent variable among 4 seasons (0.000274) as has been done elsewhere [8]. This log transformation does not affect the statistics of the non-parametric correlation test and is only relevant to the results of the multiple regression analysis and the presentation of the results.

These measures are the ratio of detected influenza numbers during the pandemic compared with the baseline. Since the baseline is two years of data, the number of detected influenza in 2020–2021 is multiplied by two in equations. Across countries, not only population is substantially different, but also surveillance systems are very different. In addition, by taking the baseline values, we can control for these two factors mostly.

We chose these 12-week periods during which influenza epidemics are common in many countries for analysis. This corresponds to the northern and southern hemisphere winter seasons. However, even if the peak of the influenza surge in a particular country does not fall within this period, it is not a problem for the purpose of the analysis of influenza is detected to some extent during these periods every year. Therefore, several countries were included in both the mid and end-season analysis. Since the exact peak of an epidemic varies slightly from year to year, even within a given country, and in some cases, there are multiple peaks within a year. It is not practical to define an analysis period based on the peak of detection for each country. The list of countries included in each analysis was provided in Supplemental Table S1. 

We chose to include two years of data as a baseline because some developing countries lack at least one year of data (especially for older data), and the more we try to include data, especially from older years, the more data we lose.

The following conditions must be met for each country to be included in the analysis of the data downloaded from the abovementioned HP [6] in June 2022.

(a) The country has detected at least 8 influenza cases in both years of the reference periods (for mid-season analyses, 2018 mid-season and 2019 mid-season; for end-season analyses, 2017 end-season and 2018 end-season).
(b) The country has data for at least 8 data of influenza subtype information (there are 7 types of influenza subtype information in each week) in all three periods of the two years of the reference periods and the 12 weeks of each analyzed period of the pandemic.
(c) The number of influenza cases detected in the reference period is at least one-tenth of the number of influenza cases detected in the other season (i.e., if 300 cases of influenza were detected in the 2-year reference period of the end-season, but only 20 cases were detected in the 2-year reference period of the mid-season, the country’s data will not be used in the mid-season analysis).
(d) The country has all the information on the mask use ratio, stringency index, and social distancing index for the relevant pandemic analysis period.

### 2.2. Mask Use

To assess each country’s mask use, data from the University of Washington Institute for Health Metrics and Evaluation (IHME) COVID-19 model site [9], which estimates daily compliance of mask use from Premise, the Facebook Global Symptom Survey (University of Maryland), the Kaiser Family Foundation, and the YouGov Behavior Tracker Survey, were utilized. Mask use is defined as the ratio of people who always wear masks in public settings and changes from 0 to 1.

The methodology for investigating mask use rates is detailed in the following study [10]. Different survey methods were used in different regions, and it is not certain that the methods so obtained have the same meaning. We see no reason to believe that such differences are systematically related to the outcomes of the study and influence the results. Nonetheless, we believe this is a limitation of the study.

### 2.3. Social Distancing

The assessment of social distancing was based on the reduction in human contact relative to background levels for each location quantified by cell phone mobility data collected from IHME. Time series on human social distancing was estimated using Gaussian process regression from data on cell phone users provided by Facebook, Google, Descartes Labs, Safegraph, and Baidu, as well as data on physical distance obligations. The Social Distancing Index, where 0 means no change, has been observed to vary from −65.8 to 83.5 on average over the period analyzed in this study across countries.

### 2.4. Stringency Index

The stringency index, the Oxford COVID-19 government response tracker, was used to assess the policy to control the pandemic [11]. The nine metrics used to calculate the Stringency Index are school closures, workplace closures, cancellation of public events, restrictions on public gatherings, closures of public transport, stay-at-home requirements, public information campaigns, restrictions on internal movement, and international travel controls.

### 2.5. Statistical Analyses of Simple Correlation Analyses

Simple correlation analyses were performed using Predictive Analysis Software, version 22.0.0 (SPSS Inc., Chicago, IL, USA; 2010). Spearman’s non-parametric correlation tests were performed between each infection control measure and the change in the influenza cases detected. 

### 2.6. Statistical Analyses of Multiple Regression Analyses Adjusting for Change of Number of Processed Specimens

Multiple regression analyses were performed using R software version 4.0.1 [12]. 

Multiple regression analyses were performed to assess the association of influenza levels with mask use ratio, social distancing index, and stringency index measures after adjusting for these measures each other as well as changes in the number of processed specimens. Multiple regression analyses were conducted for 4 different time periods: 2020 mid-season, 2020 end-season, 2021 mid-season, and 2021-end-season. The independent variables included the mask use ratio, social distancing index, stringency index, and the log-transformed ratio for the number of processed specimens compared with the baseline periods (e.g., 2 × the number of processed specimens in 2020 mid-season/(the number of processed specimens in 2019 mid-season + the number of processed specimens in 2018 mid-season) in the analysis of 2020 mid-season. The information on the number of processed specimens was not complete, and there was a lack on several occasions, and how we treat these were provided in Supplemental Methods.

*P*-values were assessed by permutation (5000 iterations) based on multiple regression analyses using the ImPerm package [13] and R software. The expression for each test, as exemplified by the case of the 2020 mid-season, is as follows:
Result_x <- lmp(log_influenza_detection_ratio_2020_mid ~ mask_use_ratio_2020_mid + social_distance_2020_mid + stringency_index_2020_mid + log_ratio_of_number_of_specimens_processed_2020_mid, datasetname_y, seqs = TRUE) summary (Result_x)

Note this is an example of R code for the permutation test for the multiple regression analysis with the log-transformed influenza detection ratio of the mid-season of 2020 (compared with baseline periods) as the dependent variable, and mask use ratio, stringency index, social distancing index and the log-transformed ratio of the specimens processed compared with the baseline periods during the same period as the independent variables. The dependent variable and all independent variables in each multiple regression analysis were subjected to the permutation tests for each analysis.

Permutation analyses were conducted nineteen times, while conducting the analyses 19 times led to more stable results. This number was chosen empirically, and the median *p*-value was used for comparisons. For all analyses, results with a threshold of *p* < 0.05 (one-sided in the case of infection control measures and two-sided in the case of the variable of the ratio of the number of specimens processed) after correcting for the false discovery rate using a graphically sharpened method [14] were considered statistically significant.

### 2.7. Additional Analyses of Associations between Positivity Rate and NPIs

We then compared the nature of the influenza positivity rate with the results of the main analysis, which used a measure of how many more positive counts were during the pandemic compared to the baseline. The measure of positivity rate was not used in the main analysis as it may not be a sensitive indicator if testing itself had been reduced due to the apparent absence of influenza during the pandemic. The positivity rate is calculated by using the measure for the number of processed specimens used in the multiple regression analysis above as the denominator and the number of positives for the corresponding data type as the numerator. Only country regions where data on the sum of the number of positive cases of all data types existed on the website where we downloaded the data of positive cases in the main analysis [6] are included in the analysis.

As in the main analysis, “2 × positivity rate during the relevant period of the pandemic/average positivity rate during the relevant period of the baseline two years” was then calculated. Furthermore, as in the main analysis, the lowest non-zero value in the four seasons of this value was added to the respective country value and then log-transformed. The correlations between this log-transformed value and the NPI measures were then analyzed in the same way as in the main analysis. These were analyzed by Spearman’s non-parametric simple correlation analysis.

## 3. Results

### 3.1. Analyses of 12 Weeks from Epidemiological Week 23 in the 2020 Period (Analysis of Countries Mainly in the Southern Hemisphere and around the Equator)

**Figure 1:**
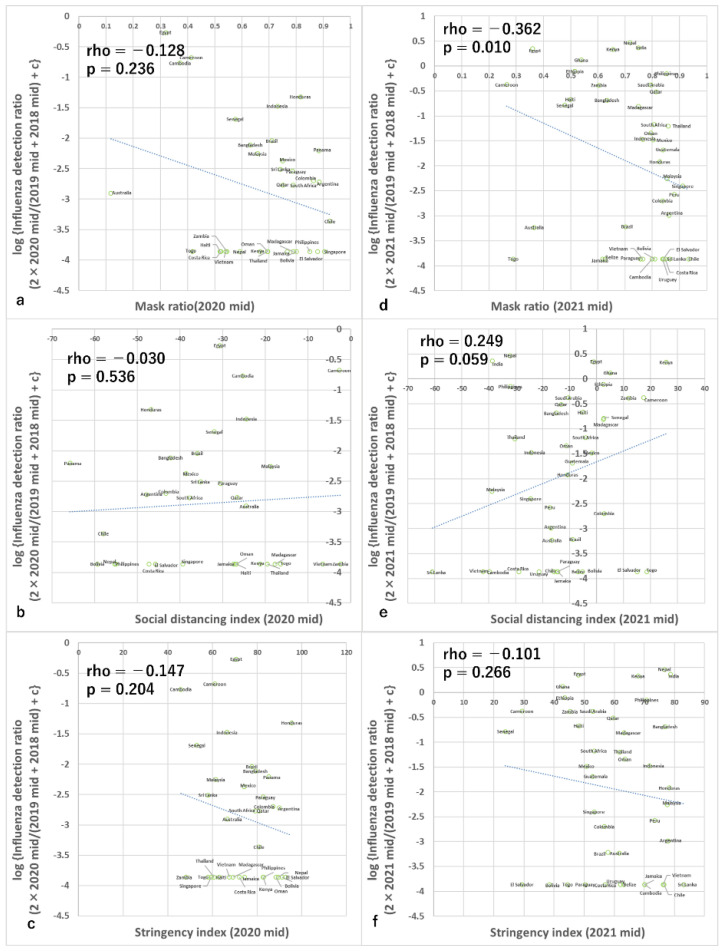
Association between infection control measures and influenza reduction in the mid-seasons. Association between influenza detection rate and (**a**) mask use in 2020, (**b**) social distancing index in 2020, (**c**) stringency index in 2020, (**d**) mask use in 2021, (**e**) social distancing index in 2021, and (**f**) stringency index in 2021. c in the y-axis is 0.0137. The *p*-value is Spearman’s simple correlation test with a one-tailed test. Rho is the correlation coefficient of non-parametric correlation analyses. Note the many data points of lowest value of y axis in all the panels correspond to no detection of influenza during the period according to the conversion equation.

Data from 34 countries met the criteria for analysis during this period. An analysis of countries in the Southern hemisphere and around the equator during the summer of 2020 found that most analyzed countries (31 out of 34 countries) detected fewer than 10% cases of influenza compared to the previous years. Most countries also demonstrated both a high mask-use ratio and a decline in the human social distancing index, with the stringency index concentrated at high values (Figure 1). All countries demonstrated a stringency index >45, 27 of 34 countries demonstrated a social distancing index <−20, and 23 countries demonstrated a mask use ratio >0.6. Therefore, this period was considered to be unsuitable for analysis due to the low variation of both the detected ratio of influenza and NPI, particularly the former (meaning almost all countries took drastic measures, and in almost all countries, influenza was detected little).

However, exploration analyses were conducted, and both the simple correlation analysis and the multiple regression analysis showed no association of mask use ratio, social distancing index, or stringency index with the detection rate for influenza in each country (Table 1 and Table 2). Furthermore, during this period, the correlations among three infection control measures were all from −0.587 to +0.669 (rho and Spearman’s non-parametric correlation test) and were all high (Supplemental Table S2), but the variance inflation factors were all <3 which is the criterion for multicollinearity.

### 3.2. Analyses of 12 Weeks from Epidemiological Week 49 in 2020 (Analysis of Countries Mainly in the Northern Hemisphere and around the Equator)

**Figure 2:**
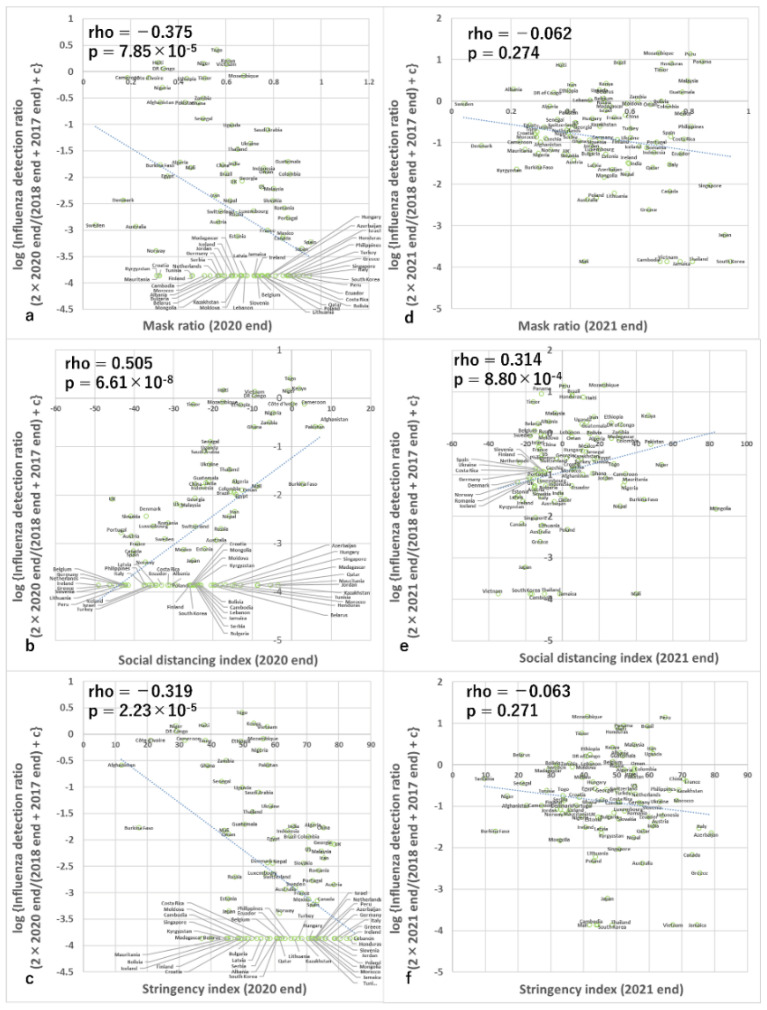
Association between infection control measures and influenza reduction in the end-seasons. Association between influenza detection rate and (**a**) mask use in 2020, (**b**) social distancing index in 2020, (**c**) stringency index in 2020, (**d**) mask use in 2021, (**e**) social distancing index in 2021, and (**f**) stringency index in 2021. c in the y-axis is 0.0137. The *p*-value is Spearman’s simple correlation test with a one-tailed test. Rho is the correlation coefficient of non-parametric correlation analyses. Note the many data points of the lowest value of the y-axis in all the panels correspond to no detection of influenza during the period according to the conversion equation.

**Figure 3:**
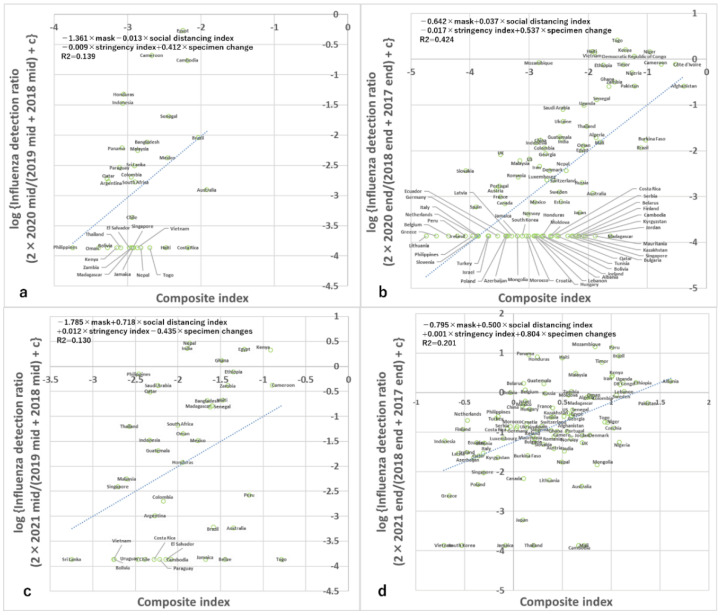
Association between the composite index of infection control measures and influenza reduction in all four seasons. Association between influenza detection rate and the composite index of mask use, social distancing index, stringency index, and number of weeks with data in (**a**) 2020 mid-season, (**b**) 2020 end-season, (**c**) 2021 mid-season, and (**d**) 2021 end-season. c in the y-axis is 0.0137. Note the many data points of the lowest value of the y-axis in panel b correspond to no detection of influenza during the period according to the conversion equation.

Data from 97 countries met the criteria for analysis during this time period. Simple correlation analyses (Spearman and non-parametric) using data from the 12 weeks of epidemiological week 49 in 2020, primarily from countries in the Northern Hemisphere and around the equator, found that the ratio of detection of influenza was negatively correlated with the rate of mask use, positively correlated with the human social distancing index, and negatively correlated with the stringency index (Figure 2, Table 1). These relationships were also significant in multiple regression analyses that included these variables and the ratio of specimens processed during the period compared with baseline periods, but the association with the stringency index on multiple regression analysis was only marginally significant (Figure 3, Table 2). Furthermore, during this period, the correlations among 3 infection control measures were all from −0.536 to +0.446 (rho and Spearman’s non-parametric correlation test) and were all relatively high (Supplemental Table S3), but the variance inflation factors were all <3 which is the criterion for multicollinearity.

### 3.3. Analyses of 12 Weeks from Epidemiological Week 23 in 2021 (Analysis of Countries Mainly in the Southern Hemisphere and around the Equator)

Data from 41 countries met the criteria for analysis during this period. Simple correlation analyses (Spearman and non-parametric) using data from 12 weeks from epidemiological week 23 in 2021, primarily from countries in the Southern Hemisphere and around the equator, found that the ratio of detection of influenza was negatively correlated with the ratio of mask use, but did not correlate significantly with the human social distancing index or the stringency index (Figure 1, Table 1). Multiple regression analyses that included these variables and the ratio of specimens processed during the period compared with baseline periods also found similar results (Figure 3, Table 2). Furthermore, during this period, the correlations among three infection control measures were all from −0.723 to +0.420 (rho and Spearman’s non-parametric correlation test) and were all high (Supplemental Table S4), but the variance inflation factors were all <3 which is the criterion for multicollinearity.

### 3.4. Analyses of 12 Weeks from Epidemiological Week 49 in 2021 (Analysis of Countries Mainly in the Northern Hemisphere and around the Equator)

Data from 97 countries met the criteria for analysis during this period. Simple correlation analyses (Spearman and non-parametric) using data from 12 weeks from epidemiological week 49 in 2021, primarily from countries in the Northern Hemisphere and around the equator, found that the ratio of detection of influenza was significantly and negatively correlated with the ratio of mask use, significantly and positively correlated with the human social distancing index, but did not significantly correlate with the stringency index (Figure 2, Table 1). Multiple regression analyses that included these variables and the ratio of specimens processed during the period compared with baseline periods also demonstrated the same results (Figure 3, Table 2). Furthermore, during this period, the correlations among three infection control measures were all from −0.256 to +0.362 (rho and Spearman’s non-parametric correlation test) (Supplemental Table S5), but the variance inflation factors were all <3 which is the criterion for multicollinearity. 

### 3.5. Additional Analyses of Associations between Positivity Rate and NPIs

The results are shown in Table 3. Basically, the significant and non-significant results of the simple correlation analyses using the measure of positivity rate and the measure of positive case number are similar, and our conclusions and discussions were not affected by the results of these additional analyses.

### 3.6. Supplemental Analyses for the Associations between the Outbreaks of COVID-19 and Influenza

This study does not include the factor of the spread of COVID-19 or viral interference. There are multiple reasons for this. First, this hypothesis is already suggested unlikely to explain the disappearance and resurgence of influenza during this pandemic, as many countries and areas temporarily experienced the disappearance of influenza before the serological spread of COVID-19 [3]. Further, many countries are now experiencing simultaneous outbreaks of COVID-19 and influenza while infection control measures are being weakened [5].

In addition, there is a significant discrepancy between the number of positives of COVID-19 and the number of actual cases among countries, especially in many developing countries, and there is no baseline to correct for this, making it difficult to assess spreads of virus by country. Antibody positivity rates are a reliable indicator of a spread if measured accurately, but antibody positivity rates and assessments of change are not available for most countries for the time period covered in this study. The number of deaths due to COVID-19 seems to be a somewhat reliable indicator of an outbreak in developed countries, but this is no longer the case since the start of vaccination.

We do not believe that a good indicator of differences in outbreaks of COVID-19 by country is available, but as a supplementary analysis, we examined the correlation between the detection ratio of influenza cases and the number of positive cases of COVID-19 and deaths due to COVID-19 during the analysis periods among countries with a low discrepancy between excess deaths and deaths due to COVID-19 (assuming such countries detect most of the deaths due to COVID-19).

As criteria for inclusion in this analysis, we selected countries where the ratio of cumulative excess deaths to cumulative deaths of COVID-19 was 0.4 or higher during the pandemic period in our world in data until February 2022 or where the cumulative excess deaths were negative until that time. We did not analyze the 2020 mid-season and 2021 mid-season. This is because few of these countries meet the above criteria during these periods.

For the number of positive cases of COVID-19, we used the number of positives per million people in 90 days from 1 December 2020, or 2021, or the number of deaths due to COVID-19 per million people in 90 days from December 15 for the data. Data were from Our world in data [11].

Results showed that the influenza detection ratio was not significantly correlated with either the number of positive cases of COVID-19 per million or the number of deaths due to COVID-19 per million in the 2020 end-season (positive cases: *p* = 0.300 (two-sided), rho = 0.155, N = 47, deaths: *p* = 0.999 (two-sided), rho = 2.46 × 10^−4^, N = 47) nor in the 2021 end-season (positive cases: *p* = 0.381 (two-sided), rho = −0.129, N = 48, deaths: *p* = 0.045 (two-sided), rho = 0.760, N = 48). Future studies using seropositivity before vaccination starts may help understand the spread of COVID-19 and the return of influenza.

## 4. Discussion

This study examines the country-specific factors involved in the disappearance of influenza during the COVID-19 pandemic. In mid-2020, most equatorial and Southern hemisphere countries had high levels of infection control measures in place, either mask use, reduced human social distancing index, or a high stringency index, and in most countries, influenza was drastically reduced compared to previous years. The results of the analysis using end-2020, end-2021, and mid-2021 were in part consistent with this hypothesis, with high mask use being associated with the disappearance of influenza in all seasons. The season of mid-2020, while not statistically significant, showed a tendency for a negative correlation between mask use and influenza detection ratio. A low social distancing index was strongly associated with influenza disappearance in one season (end of 2020) and weakly associated in another (end of 2021). A high stringency index was weakly associated with influenza disappearance in the end-2020 season.

The disappearance of influenza was significantly associated with a higher local mask ratio in end-2020, mid-2021, and end-2021. Despite the low variance of the influenza detection rate in the mid-2020 season, mask use also showed a tendency toward a negative association with the influenza detection rate during this season. Previous meta-analyses were unable to detect significant differences with regard to the effects of mask use on influenza transmission or spread [15]. However, a number of recent meta-analyses of randomized controlled trials of the effects of masks used on the suppression of transmission or spread of respiratory viruses [16] supported the effectiveness of masks. Differences in the outcomes of various studies may be caused by the need for statistical power when analyses are limited to the influenza virus, as the recent intervention study showed that an enormous sample size is required to detect the effects of mask use [17]. The effectiveness of masks was also noted in a meta-analysis of case-control studies and cohort studies [18]. A review of multiple real-world mask studies against novel coronaviruses also supported the effectiveness of masks [19]. Masks were shown to decrease aerosol shedding, including influenza particles [20], while influenza is considered to be transmitted only in part through aerosol [21]. The results of this study are consistent with the overwhelming evidence for the effectiveness of masks that have accumulated during the COVID-19 pandemic.

The stringency index, an integrated index of the various degrees of regulation, was associated only with the disappearance of influenza in end-2020. In the simple non-parametric correlation, the correlation was strong, but the relationship was weaker when mask use and human social distancing were controlled for in the multiple regression analysis. No significant results were found in the analyses of the mid- and end-2021, irrespective of whether mask use or social distancing was corrected. Attention should be paid to the marginally statistically significant results of the analyses of end-2020, as they were not reproducible in the analyses of 2021. One of the reasons for the lack of consistent results is that the stringency index covers a wide range of measures, such as school closures, workplace closures, cancellation of public events, restrictions on public gatherings, closure of public transport, stay-at-home requirements, public information campaigns, restrictions on internal movement, and international travel controls. Some of these may be effective, and some may not, which may explain the weak association with the influenza surge when evaluated as an overall index. For example, some studies suggest that school closures are not effective against the spread of COVID-19 [22]. Some of these were specifically targeted to SARS-CoV-2 or administered specifically in response to COVID-19. International travel may also not be as effective in countries where travel to and from other countries is relatively easy such as the European Union. It would be preferable to evaluate each measure individually in the future, as has been in previous studies. In addition, as for the stringency index, the degree to which people follow the government’s recommendations or orders varied over time and per country, and from this perspective, it may not reflect people’s behaviors well, and that may lead to the results of low associations of this measure with influenza detection.

Low human social distancing indices were associated with the disappearance of influenza in the end-2020 and end-2021 seasons. Previously, a low human mobility index was shown to be associated with the lower spread of COVID-19 in its early phase between countries [23]. It was also shown that factors related to reduced human flow, such as bans on mass gatherings, were associated with a slower spread of COVID-19 [24]. It was also shown that fixed country factors, such as the number of annual air passengers and the degree to which populations are concentrated in urban areas, were associated with the spread of COVID-19 [24]. Individual analyses also showed that people with a lower social distance from others were more likely to have COVID-19 [25]. The results of the present study are in line with this body of work and are consistent with the hypothesis that human flow and social distancing are also important in reducing the spread of influenza.

There are several limitations to this study. First, it was not possible to assess the effects of handwashing, which has been shown to be effective in previous RCTs, due to a lack of information. Another general limitation of such country-specific analyses is that neighboring countries are inextricably linked with respect to multiple factors. It is, therefore, possible that the correlations noted may be, in fact, spurious. For example, people who use masks are more likely to wash their hands at the same time, which may have been more effective. In this study, information on hand washing was not available from many countries. In order to resolve these concerns, intervention studies using a large sample size are required, as has been done in the case of COVID-19. Other than that, factors that have been suggested to be effective NPIs, such as travel bans, school closures, and mass gathering bans, were not evaluated separately in this study [3,5,24]. These overlap in part with the social distancing index and the stringency index but do not fully match. The impact of these individual policies will need to be assessed by future studies. Another limitation of this study is that countries may have variations on how much influenza vaccine they had before the pandemic and how much they continued to receive during the pandemic, but unlike the NPI information, that information is not available for many countries. Since influenza was prevalent every year before the pandemic throughout the world, including in countries where influenza vaccine was frequently administered, one would expect that influenza vaccination would not have a critical enough impact to eliminate the spread of influenza, but this is speculation. The impact of these factors should be analyzed in the future when vaccination information is available for many countries before and after a pandemic. Finally, for practical reasons, the NPI information and influenza detection information used in this analysis are only available at the country level in most countries. In reality, however, there may have been variations in NPI policies within a country, especially in larger countries.

In conclusion, this study found that influenza reduction was consistently related to a higher mask-use ratio across three of the four analyzed seasons. In the winter season in the Northern hemisphere, a low human social distancing index was consistently related to the reduction in influenza. These results may suggest that a surge of influenza is controllable through effective preventive measures and should be put into place during a possible pandemic of novel influenza viruses. 

## Figures and Tables

**Table 1:** Spearman’s simple correlations between influenza detection ratio and each control measure. Upper: correlation coefficient (rho), Lower: (*p* values).

	Mask Ratio	Social Distancing Index	Stringency Index
2020 mid	−0.128 0.236	−0.030 0.934	−0.117 0.255

2020 end	−0.375 7.85 × 10^−5^	0.505 6.61 × 10^−8^	−0.402 2.23 × 10^−5^

2021 mid	−0.362 0.010	0.249 0.059	−0.101 0.266

2021 end	−0.062 0.274	0.314 8.80 × 10^−4^	−0.063 0.271


**Table 2:** Multiple regression analyses for the associations between each measure and influenza detection. (upper left: uncorrected *p* value, upper right: corrected *p* value, lower left: unstandardized beta, lower right: standardized beta).

	2020 Mid	2020 End	2021 Mid	2021 End
Mask	0.079, 0.061 −1.361, −0.238	0.0001, 0.0002 −0.642, −0.092	0.039, 0.034 −1.785, −0.198	0.035, 0.034 −0.795, −0.131
Social distancing index	0.684, 0.314 −0.013, −0.192	0.0001, 0.0002 0.037, 0.354	0.220, 0.140 0.019, 0.219	0.030, 0.034 0.010, 0.179
Stringency index	0.328, 0.191 −0.009, −0.111	0.035, 0.034 −0.017, −0.183	0.718, 0.314 0.012, 0.116	0.500, 0.250 0.001, 0.013
Specimen number change	0.166, 0.116 0.412, 0.256	0.004, 0.007 0.537, 0.244	0.375, 0.202 0.435, 0.160	0.0001, 0.0002 0.804, 0.375

*p* values for each control measure are one-sided, while those of the specimen number changes are two-sided.

**Table 3:** Spearman’s simple correlations between percentage of influenza cases compared to baseline cases (upper, main analyses)/percentage of positivity rates during the pandemic compared to baseline positivity rates (lower) and each control measure. Left: correlation coefficient (rho), Right: (*p* values).

		Mask Ratio	Social Distancing Index	Stringency Index
2020 mid	Case	−0.128, 0.236 −0.140, 0.218	−0.030, 0.934 0.023, 0.450	−0.117, 0.255 −0.056, 0.378
	Positivity rate			
2020 end	Case	−0.375, 7.85 × 10^−5^ −0.348, 2.38 × 10^−4^	0.505, 6.61 × 10^−8^ 0.475, 4.47 × 10^−7^	−0.402, 2.23 × 10^−5^ −0.395, 3.1 × 10^−5^
	Positivity rate			
2021 mid	Case	−0.362, 0.010 −0.362, 0.010	0.249, 0.059 0.267, 0.045	−0.101, 0.266 −0.098, 0.271
	Positivity rate			
2021 end	Case	−0.062, 0.274 −0.004, 0.485	0.314, 8.80 × 10^−4^ 0.397, 2.85 × 10^−5^	−0.063, 0.271 −0.097, 0.172
	Positivity rate			

## Data Availability

Relevant data can be downloaded from the following sites: influenza (https://apps.who.int/flumart/Default?ReportNo=7 or https://app.powerbi.com/view?r=eyJrIjoiNjViM2Y4NjktMjJmMC00Y2NjLWFmOWQtODQ0NjZkNWM1YzNmIiwidCI6ImY2MTBjMGI3LWJkMjQtNGIzOS04MTBiLTNkYzI4MGFmYjU5MCIsImMiOjh9); mask use ratio, (https://covid19.healthdata.org/); social distance index, (https://covid19.healthdata.org/); stringency index, (https://ourworldindata.org/explorers/coronavirus-data-explorer). Processed data that was used in the analysis was provided through the supplementary online material.

## Supplementary Materials

The following supporting information can be downloaded at: https://www.mdpi.com/article/10.3390/v15010223/s1. Table S1: The list of countries included in the analysis and the weeks with the highest number of influenza cases detected in the years used as reference. Table S2: Simple correlation matrix of covariates during the 2020 mid-season (Upper line: correlation coefficient, rho, lower line: *p*-value); Table S3: Simple correlation matrix of covariates during the 2020 end-season (Upper line: correlation coefficient, rho, lower line: *p*-value). Table S4: Simple correlation matrix of covariates during the 2021 mid-season (Upper line: correlation coefficient, rho, lower line: *p*-value). Table S5: Simple correlation matrix of covariates during the 2021 end season (Upper line: correlation coefficient, rho, lower line: *p*-value). Supplementary Data: Soucce data used in the analysis.
