# Supplementary material for: Disappearance and Re-Emergence of Influenza during the COVID-19 Pandemic: Association with Infection Control Measures
Source: Viruses. 2023 Jan 13;15(1):223. doi: 10.3390/v15010223 (PMC9862942; doi:10.3390/v15010223)
Supplement: Supplementary file 1 [file viruses-15-00223-s001.zip › flurevival_SOM_virusesproof_trackchangeupdated_submitted.pdf]

Supplemental Online Material

Supplemental Methods

Handling of information on the number of specimens processed

Information on the number of specimens processed for influenza testing during each period in each country is provided by the following page [1]. However, there are some omissions in this data and this data is divided into three types of data: sentinel, non-sentinel and not defined (existence of sentinel/non-sentinel data and existence of not-defined data are mostly mutually exclusive in each week in each country), unlike the data on the total number of positive influenza cases that had been provided on the following page [2] (the total number of positive cases in the latter page should be the data on the sum of number of influenza positives of all types on the former page). This subsection therefore explains how we have treated these data.

Basically, for most countries, we used all available data to produce the total number of specimens processed. However, there were some exceptions. Jamaica was excluded from the analyses using the data of the number of processed specimens for the 2020 mid season because data on the number of specimens processed were not available for the 2020 mid season. Since no data of the number of specimens processed exist in the non-sentinel data for England and the Netherlands, we used only sentinel-data for these regions. The other three UK regions have both sentinel and non-sentinel data, so we used both data for those three regions and combined the data from the four regions to calculate the positivity rate for each period. We used only non-sentinel data for Hungary because there was a period in the Hungarian data when the number of specimens processed did not exist in the end season in the sentinel data. Although the Japanese data did not contain data on the number of specimens processed, information on the number of negative and positive specimens was available, so the sum of these was treated as the number of specimens.

Table S1. The list of countries included in the analysis and the weeks with the highest number of influenza cases detected in the years used as reference.

country	Analysis of mid-2020	Analysis of mid-2021	Analysis of end-2020	Analysis of end-2021	Influenza peak week from w36 in 2017 to w35 in 2018	Influenza peak week from w36 in 2018 to w35 in 2019
Afghanistan			O	O	1	4
Albania			O	O	6	5
Algeria			O	O	5	8
Argentina	O	O			35	27
Australia	O	O	O	O	36	26
Austria			O	O	6	7
Azerbaijan			O	O	3	3
Bangladesh	O	O			29,31	24
Belarus			O	O	11	6
Belgium			O	O	7	7
Belize		O			31	45
Bolivia	O	O	O	O	16	23
Brazil	O	O	O	O	20	24
Bulgaria			O	O	4	4
Burkina Faso			O	O	5	50
Cambodia	O	O	O	O	40	25
Cameroon	O	O	O	O	42	46
Canada			O	O	6	1
Chile	O	O			33	24
China			O	O	3	3
Colombia	O	O	O	O	23	29
Costa Rica	O	O	O	O	41,45	24
Côte d'Ivoire			O		46	24,29
Croatia			O	O	2	3
Czechia				O	8	5
Democratic Republic of Congo			O	O	49	52
Denmark			O	O	8	6

Ecuador			O	O	4	1
Egypt	O	O	O	O	10	49
El Salvador	O	O			35	44
Estonia			O	O	10	6
Ethiopia		O	O	O	2	32
Finland			O	O	6	7
France			O	O	1	6
Georgia			O	O	6	52
Germany			O	O	9	6
Ghana		O	O	O	51	40
Greece			O	O	9	4,5
Guatemala		O	O	O	11	14,31
Haiti	O	O	O	O	38,31	13
Honduras	O	O	O	O	18,19	34
Hungary			O	O	7	6
Iceland			O	O	11	10
India		O	O	O	1	7
Indonesia	O	O	O	O	45	5
Iran			O	O	52	2
Ireland			O	O	3	6
Israel			O	O	2	5
Italy			O	O	2	6
Jamaica	O	O	O	O	7	9
Japan			O	O	3	4
Jordan			O	O	52	4
Kazakhstan			O	O	12	4
Kenya	O	O	O	O	31	29,31
Kyrgyzstan			O	O	7	3
Latvia			O	O	11	6
Lebanon			O	O	11	8
Lithuania			O	O	6	4
Luxembourg			O	O	10	7
Madagascar	O	O	O	O	8	4
Malaysia	O	O	O	O	10	25
Mali			O	O	6	41
Mauritania			O	O	40,7	2

Mexico	O	O	O	O	4	2
Mongolia			O	O	3	3
Morocco			O	O	4	6
Mozambique			O	O	44	12
Nepal	O	O	O	O	36	4
Netherlands			O	O	9	8
Niger			O	O	8	49
Nigeria			O	O	3	45,27
Norway			O	O	7	7
Oman	O	O	O	O	44	46
Pakistan			O	O	4	6
Panama	O			O	43	25
Paraguay	O	O			32	23
Peru		O	O	O	26	33
Philippines	O	O	O	O	32	2
Poland			O	O	9	6
Portugal			O	O	6	6
Qatar	O	O	O	O	49	44
South Korea			O	O	3	51
Moldova			O	O	10	6
Romania			O	O	7	4
Russia			O	O	13	8
Saudi Arabia		O	O		47	47
Senegal	O	O	O	O	43	39
Serbia			O	O	5	6
Singapore	O	O	O	O	6	29
Slovakia			O	O	9	8
Slovenia			O	O	10	5
South Africa	O	O			24	24
Spain			O	O	1	6
Sri Lanka	O	O			27	50
Sweden			O	O	7	6
Switzerland			O	O	1	7
Thailand	O	O	O	O	36	9
Timor			O	O	10	15
Togo	O	O	O	O	46	43,47

Tunisia			O	O	52	10
Turkey			O	O	3	2
Uganda			O	O	49	28,34
Ukraine			O	O	8	3
Urugay		O			34	37,31
United Kingdom			O	O	2	6
United States			O	O	5	8
Vietnam	O	O	O	O	38	45
Zambia	O	O	O	O	16	34

Table S2. Simple correlation matrix of covariates during the 2020 mid-season (Upper line: correlation coefficient, rho, lower line: p value).

	Mask use ratio	Social distancing index	Stringency index	Specimens processed
Mask use ratio	1	-0.613<br>1.48×10^{-4}	0.581<br>3.97×10^{-4}	-0.287<br>0.105
Social distancing index		1	-0.673<br>1.80×10^{-5}	0.205<br>0.252
Stringency index			1	-0.269<br>0.130
Specimens processed				1

Table S3. Simple correlation matrix of covariates during the 2020 end season (Upper line: correlation coefficient, rho, lower line: p value).

	Mask use ratio	Social distancing index	Stringency index	Specimens processed
Mask use ratio	1	-0.452	0.446	-0.258
		3.31×10^{-6}	4.80×10^{-6}	0.011
Social distancing index		1	-0.536	0.232
			1.55×10^{-8}	0.022
Stringency index			1	-0.234
				0.021
Specimens processed				1

Table S4. Simple correlation matrix of covariates during the 2021 mid-season (Upper line: correlation coefficient, rho, lower line: p value).

	Mask use ratio	Social distancing index	Stringency index	Specimens processed
Mask use ratio	1	-0.528	0.420	-0.440
		3.84×10^{-4}	6.30×10^{-3}	0.004
Social distancing index		1	-0.723	0.172
			9.59×10^{-8}	0.282
Stringency index			1	-0.109
				0.496
Specimens processed				1

Table S5. Simple correlation matrix of covariates during the 2021 end season (Upper line: correlation coefficient, rho, lower line: p value).

	Mask use ratio	Social distancing index	Stringency index	Specimens processed
Mask use ratio	1	-0.256	0.362	0.008
		0.011	2.69×10^{-4}	0.942
Social distancing index		1	-0.251	0.009
			0.013	0.930
Stringency index			1	-0.025
				0.804
Specimens processed				1

References

1. World_Health_Organization Influenza Laboratory Surveillance Information by the Global Influenza Surveillance and Response System (GISRS). Available online: . https://app.powerbi.com/view?r=eyJrIjoiNjViM2Y4NjktMjJmMC00Y2NjLWFmOWQtODQ0NjZkNWM1YzNmIiwidCI6ImY2MTBjMGI3LWJkMjQtNGIzOS04MTBiLTNkYzI4MGFmYjU5MCIsImMiOjh9 (acessed on 1st June 2022)
2. World_Health_Organization Influenza Laboratory Surveillance Information by the Global Influenza Surveillance and Response System (GISRS). https://apps.who.int/flumart/Default?ReportNo=7 (acessed on 1st June 2022)
